# Allopolyploidy: An Underestimated Driver in *Juniperus* Evolution

**DOI:** 10.3390/life13071479

**Published:** 2023-06-30

**Authors:** Perla Farhat, Sonja Siljak-Yakovlev, Najat Takvorian, Magda Bou Dagher Kharrat, Thierry Robert

**Affiliations:** 1 Laboratoire Biodiversité et Génomique Fonctionnelle, Faculté des Sciences, Université Saint-Joseph, Campus Sciences et Technologies, Mar Roukos, Mkalles, BP, 1514 Riad el Solh, Beirut 1107 2050, Lebanon; perla.farhat@ceitec.muni.cz (P.F.); magda.boudagher@efi.int (M.B.D.K.); 2 Ecologie Systématique Evolution, Univ. Paris-Sud, CNRS, AgroParisTech, Université Paris-Saclay, 91190 Gif-sur-Yvette, France; najat.takvorian@sorbonne-universite.fr; 3 CEITEC—Central European Institute of Technology, Masaryk University, Kamenice 5, 625 00 Brno, Czech Republic; 4 Faculté des Sciences et Ingénierie, Sorbonne Université, UFR 927, 4 Place Jussieu, 75252 Paris, France; 5 European Forest Institute, Mediterranean, Sant Pau Art Nouveau Site, St. Antoni M. Claret, 167, 08025 Barcelona, Spain

**Keywords:** AFLP, conifers, genetic admixture, hybridization, *Juniperus*, polyploidy

## Abstract

Allopolyploidy is considered as a principal driver that shaped angiosperms’ evolution in terms of diversification and speciation. Despite the unexpected high frequency of polyploidy that was recently discovered in the coniferous genus *Juniperus*, little is known about the origin of these polyploid taxa. Here, we conducted the first study devoted to deciphering the origin of the only hexaploid taxon in *Juniperus* along with four of its closely related tetraploid taxa using AFLP markers with four primers combinations. Phylogenetic analysis revealed that the 10 studied species belong to 2 major clusters. *J. foetidissima* appeared to be more related to *J. thurifera*, *J. sabina*, and *J. chinensis*. The Bayesian clustering analysis showing a slight variation in genetic admixture between the studied populations of *J. foetidissima*, suggesting an allopolyploid origin of this species involving *J. thurifera* and *J. sabina* lineages, although a purely autopolyploidy origin of both *J. thurifera* and *J. foetidissima* cannot be ruled out. The admixed genetic pattern revealed for *J. seravschanica* showed that the tetraploid cytotypes of this species originated from allopolyploidy, whereas no clear evidence of hybridization in the origin of the tetraploid *J. thurifera* and *J. chinensis* was detected. This study provides first insights into the polyploidy origin of the *Sabina* section and highlights the potential implication of allopolyploidy in the evolution of the genus *Juniperus*. Further analyses are needed for a more in-depth understanding of the evolutionary scenarios that produced the observed genetic patterns.

## 1. Introduction

Natural hybridization and polyploidy have long been considered as partners in plant evolution and promoters of biodiversity [1]. Allopolyploidy is when genome duplication involves the genomes from diverged species through hybridization, inducing diversification and speciation. Indeed, allopolyploidy is a common pathway for hybrid speciation [2,3,4]. It has been well documented in angiosperms, yet overlooked in gymnosperms (except *Ephedra* L. [5,6]). Recently, an unexpected frequency of polyploid species (15% of natural taxa) was determined in the coniferous genus *Juniperus* L. It was suggested that at least ten polyploidy events have occurred in the course of *Juniperus* evolution [7]. All polyploid *Juniperus* taxa are distributed in the old world and mainly in the Mediterranean region [7]. *Juniperus* is the most diversified genus in the Cupressaceae family and the second in the conifers after *Pinus* L., with approximately 75 species widely distributed in the northern hemisphere from sea level until the tree line, except for one species. Only *Juniperus procera* Hochst. ex Endl. is restricted to the Southern Hemisphere [8]. This monophyletic genus is divided into 3 monophyletic sections, *Caryocedrus* (1 species, *Juniperus drupaceae* Labill.), *Juniperus* (13 species), and *Sabina* (61 species) [8]. All polyploidy cases in this genus have been observed in the *Sabina* section [7,9,10,11,12]. This section includes diploid species (2*n* = 2*x* = 22), tetraploid species (2*n* = 4*x* = 44), and just one hexaploid *Juniperus foetidissima* Willd. (2*n* = 6*x* = 66) [7,9,10,11,13]. Interestingly, *J. foetidissima* is the only identified hexaploid conifer found in the Mediterranean region [7] and only the second identified in the whole world, the first one being *Sequoia sempervirens* (D.Don) Endl. [14].

*Juniperus foetidissima* is a dioecious, conical tree reaching up to 20 m in height. Its scale leaves are thick (ca. 1.5 mm) comparing to *Juniperus* closely related species.. The seed cones, globose, 7–12 mm, maturing in 2 years, commonly contain just 1 or 2 seeds. This species is distributed in the eastern Mediterranean region, mainly on rocky high mountains. It is found on the Balkans Mountains in Albania, Greece, North Macedonia, on Caucasus Mountains, Turkey, Syria, Lebanon, southwestern Turkmenistan, Iran’s high mountains, and in an isolated population on Crimea [8,15]. The origin of *J. foetidissima*, and especially whether it is an allopolyploid or an autopolyploid, is still unclear. Molecular phylogenetic inferences have placed *J. foetidissima* in the third major clade (corresponding to the *Sabina* section) on the sister branch of the tetraploid species *J. thurifera* L. [8]. *Juniperus foetidissima* appears to be closely related to *J. excelsa* M. Bieb., *J. polycarpos* K. Koch., and *J. seravschanica* Kom. [8]. This suggests that the ancestors of some of these species could have been involved in the origin of *J. foetidissima*, if it was an allopolyploid. Nevertheless, this phylogenetic tree of *Juniperus* was built using ITS (nuclear region) and four chloroplast regions. These markers have been shown to be efficient for phylogenetic reconstructions. However, due to their relatively low polymorphism compared with other markers such as AFLP, their ability to resolve relationships between closely related species is limited [16]. The AFLP technique can generate a large number of biallelic markers, which are sampled in approximately the entire genome. They usually display strong phylogenetic signals among closely related species [17,18,19,20,21].

The first aim of this study was to decipher the origin of the hexaploid *J. foetidissima* by using AFLP markers. *Juniperus* species of *Sabina* section were selected for this study based on their belonging to the same clade of *J. foetidissima* in the phylogenetic tree [8] and based on their close geographical distribution near this species. Recently, evidence of current or past events of interspecific hybridization has been revealed between *J. sabina* and *J. thurifera* lineages [22,23,24]. Therefore, our second aim was to investigate the signals of genetic admixture and phylogenetic relationships between the chosen species in this study using AFLP markers.

## 2. Materials and Methods

### 2.1. Plant Material

The selected taxa included in this study were: *J. chinensis* L., *J. excelsa*, *J. foetidissima*, *J. phoenicea* L., *J. polycarpos*, *J. procera*, *J. sabina* var. *sabina* L., *J. sabina* var. *balkanensis* R. P. Adams and A. Tashev., *J. seravschanica*, *J. thurifera*, and *J. turcomanica* B. Fedtsch. (Table 1). *Juniperus phoenicea* was chosen as an outgroup.

In total, 147 *Juniperus* samples (fresh or dried leaves) were analyzed (details are presented in Table 1). All dried leaves samples were provided in silica gel from Baylor University Herbarium (BAYLU), except for samples of *J. thurifera* from the French Alps, which were provided dried from the herbaria of the National Alpine Botanical Conservatory (CBNA).

### 2.2. DNA Extraction

Tot section al genomic DNA was extracted via the cetyltrimethyl ammonium bromide (CTAB) method [26], according to the modifications for conifers described by Bou Dagher-Kharrat [18]. Approximately 30 mg of dried leaves was ground in a 2% CTAB solution (1.4 M NaCl, 20 mM EDTA, and 100 mM Tris-HCl; pH 8.0; 2% CTAB; and 2% polyvinylpyrrolidone (PVP)).

### 2.3. Amplified Fragment Length Polymorphism (AFLP) Analysis

The AFLP method used is based on the standard protocol [27]. To improve the reliability of AFLP data, 34 replicates of AFLP profiles were generated from independent restriction digests of the same DNA (repetition type 1) and from different DNA extractions of the same sample (repetition type 2) (Table 2). Replicates covered around 23% of the entire samples of the dataset. Additionally, 8 repetitions of the negative control (H_2_O) were included. Genomic DNA (250 ng) was digested at 37 °C for 2 h 30 min. The digestion reaction contained 5 units of EcoRI and 5 units of Tru1I restriction enzymes in 1X ligase buffer (Fermentas MBI, Burlington, ON, Canada) in a final volume of 25 μL. Then, restriction enzymes were inactivated at 70 °C for 20 min. The ligation reaction mix contained 5 pmol EcoRI adaptors and 50 pmol Tru1I adaptors, 1X ligase buffer (Fermentas MBI), 1 unit of T4 DNA ligase (Fermentas MBI), and the entire digestion product (25 μL) in a final volume of 50 μL. Ligation was performed at 20 °C for 2 h 30 min. Digested and ligated DNA was diluted 10X with TE buffer (10 mM Tris, pH 8.0; 1 mM EDTA, pH 8.0). Pre-selective amplification was carried out using a primer pair complementary to each restriction site plus one specific nucleotide at the 3′ end of each primer (Table 3). Polymerase chain reaction (PCR) was carried out in a final volume of 50 μL. In each reaction, 5 μL of diluted restriction-ligation products was added to 10 pmol of each primer, 0.2 mM of each dNTP, 2.5 mM of MgCl_2_, 1X Taq DNA polymerase buffer without MgCl_2_, and 1 unit of Taq DNA polymerase (MP Biomedicals, Santa Ana, CA, USA). The pre-selective amplification protocol involved initial denaturation at 94 °C for 1 min, followed by 20 cycles at 94 °C for 30 s, 56 °C at 1 min, 72 °C at 1 min, and a final extension at 72 °C for 5 min. To verify the pre-selective amplification success, 7 μL of the PCR products was electrophoresed on 2% agarose gel stained with GelRed at 100 V for 1 h. The pre-selective amplified PCR products were diluted 50X with ddH2O. Selective amplifications using four primers combination were performed using three labeled EcoRI primers (two primers labeled with FAM and one with PET (Table 3)) and four unlabeled Tru1I (Table 3). Selective primers were similar to the pre-selective ones with the addition of two nucleotides randomly chosen at the 3′ end of the sequences (Table 3). The selective PCR contained, for each reaction, 4 μL of the diluted pre-selective PCR product, 1X of Dream Taq buffer, 0.5 mM of MgCl_2_, 0.2 mM of each dNTPs, 0.2 µM of each selective primer, and 1 unit of DreamTaq polymerase (Thermo Fisher Scientific, Waltham, MA, USA) in a final volume of 25 μL. The PCR protocol was initial denaturation at 94 °C for 1 min, 10 cycles at 94 °C for 1 min, annealing at 65 °C to 56 °C (touchdown of 1 °C per cycle) for 1 min, 72 °C for 1 min 30 s, followed by 23 cycles at 94 °C for 30 s, 56 °C for 30 s, 72 °C for 1 min, and a final extension at 72 °C for 3 min. To check the selective PCR reactions success, 7 μL of the PCR products was electrophoresed on 2% agarose and gel stained with GelRed at 100 V for 1 h.

### 2.4. AFLP Scoring

AFLP products along with GS-500 LIZ size standard were run on a capillary sequencer (Applied Biosystems^®^ 3730XL, San Francisco, CA, USA) at GENTYANE Platform-INRA (Clermont-Ferrand, France). AFLP migration profiles were analyzed using GeneMapper v.5 (Thermo Fisher Scientific). The default AFLP settings of GeneMapper v.5 software for allele parameters, peak detection algorithm, peak quality, and quality flags were used. Fragment sizes between 50 bp and 500 bp were taken into consideration in the analysis. The allele’s bins were scored as “0” if the peak height was ≤50, “1” if the peak height was ≥100, and by a “check” for the bins with the peak heights between 50 and 100. Manual analysis of the bin was performed based on the control individuals. Each peak that was present in the negative controls was discarded from the following analysis. Secondly, for each allele, genotyping error based on repetition type 1 (repetition from same DNA and independent restriction digests) and repetition type 2 (repetition from the same sample and different DNA extractions) was estimated using the Bonin error rate [28]. Loci that showed an error rate less than 10% were taken into consideration for further analyses. All loci that showed an error rate more than 10% were discarded. Then, peaks patterns that were scored as “check” were visually examined in GeneMapper and manually edited. All loci displaying singletons were also removed from the analyses.

### 2.5. AFLP Data Analysis

The proportion of polymorphic fragments (PLPs) for each primer combination was determined using aflp-survey 1.0 software [29]. Pairwise dissimilarity indices between individuals were calculated according to Nei and Li [30]. A neighbor joining (NJ) tree was created using PAUP v.4 based on the matrix of individuals dissimilarities. A heuristic search for the best NJ tree was conducted under the optimality criterion distance (minimum evolution (ME)) with 1000 bootstrap replicates. The starting seed was automatically generated. All characters had equal weight, and the branch-swapping algorithm followed tree bisection–reconnection (TBR). The builtNJ tree was unrooted and visualized using Interactive Tree Of Life (iTOL) v5 [31].

Because the occurrence of current or past events of interspecific hybridization has recently been documented between *J. sabina* and *J. thurifera* [23,24] or has involved an ancestral lineage of these species [22,32,33], we decided to also look at signals of genetic admixture between the species within the *Sabina* section represented in our sample. To achieve this goal, a Bayesian clustering approach was carried out on the basis of the AFLP fragments on the whole dataset using tess software v. 2.3.1 [34]. This procedure allowed us to infer the number (K) of unobserved ancestral genetic clusters that best explained the sample genetic structure. When the model with admixture was chosen, the proportion of individual genomes originating from each potential ancestral genetic clusters (individual ancestry coefficients (q)) was estimated. In this case, the model took into account the possibility of historical events of genetic admixture of clusters. The statistical method implemented in tess is spatially explicit in the sense that the admixture model assumes individual genetic relatedness are spatially auto correlated in a continuous manner (isolation by distance).

A first set of 140 runs (without admixture and correlated allele frequencies model) was carried out, varying K from 2 to 15 (10 runs per value of K) in order to estimate the best K values using the deviance information criterion (DIC) as a statistical variable for model choice (Figure S1). MCMCs were carried out using a burn-in period of 70,000, followed by 120,000 iterations. The best values of K were chosen as those for which the plate with the smallest deviance information criterion (DIC) values was reached. Fifty new runs were performed for each of those K values using the admixture (CAR) model.

In order to identify potential different solutions for each K value (due to potential multimodality in the posterior distributions of the q values) and to manage the label switching among replicates in the tess outputs, clumpp v1.1.2 was used with the Greedy option [35]. The probability of each solution was estimated based on their frequency among 50 runs. Plots representing the individual genomes ancestries to the inferred ancestral genetic clusters were created using the online Structure Plot v.2.0 application. Because Bayesian clustering analyses on genotyping data always generate a background of spurious genome admixture in each individual, and because this background was mostly less than 10% in most individuals belonging to diploid species, individual ancestry coefficients less than 10% were not considered as an indication of interspecific genome admixture.

## 3. Results

### 3.1. Polymorphism of AFLP Markers between Juniperus Species 

In total, the 4 primer combinations generated 1022 loci used for the analyses. The proportion of polymorphic loci (PLPs) differed between species and primer combinations (Table 4). The PLP ranged from 38.4% (for *J. polycarpos*) to 68.5% (for *J. chinensis*) in the primer combination EcoRI-1/Tru1I-1. In the primer combination EcoRI-1/Tru1I-3, the PLP ranged from 36.2% (for *J. thurifera*) to 58.5% (for *J. chinensis*). The EcoRI-2/Tru1I-4 primer combination showed the highest PLP among all the primer combinations, with 73.1% (for *J. chinensis*); the lower PLP in this primer combination was 43.8% (for *J. thurifera*). The primer combination EcoRI-4/Tru1I-2 showed a PLP ranging from 31.2% (for *J. turcomanica*) to 59.9% (in *J. chinensis*). Interestingly, *J. chinensis* showed the highest PLP for all primer combinations despite the low sample size analyzed (only nine individuals).

This observation deserves to be explored on the basis of a more representative sampling scheme.

### 3.2. Phylogenetic Relationships among Juniperus Taxa

**Figure 1:**
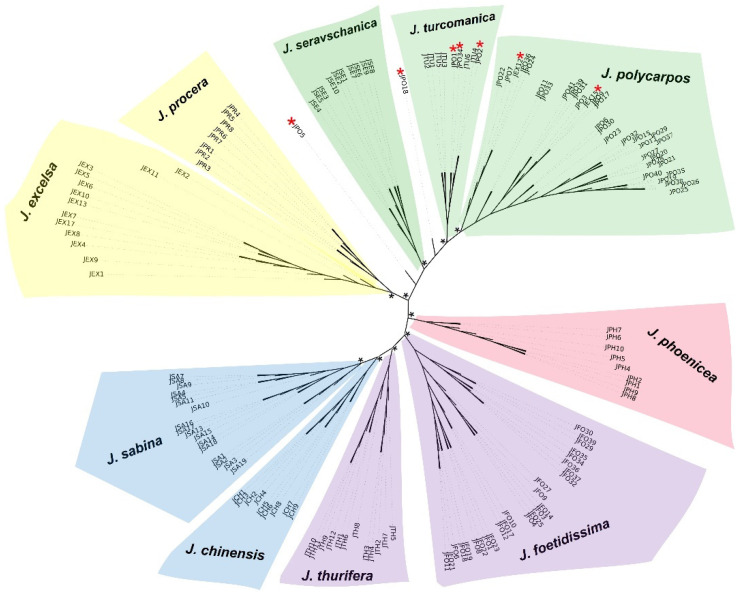
NJ phylogenetic distance, unrooted tree including ten *Juniperus* species. Species abbreviations: JEX: *J. excelsa*, JPR: *J. procera*, JSE: *J. seravschanica*, JTU: *J. turcomanica*, JPO: *J. polycarpos*, JPH: *J. phoenicea*, JFO: *J. foetidissima*, JTH: *J. thurifera*, JCH: *J. chinensis*, and JSA: *J. sabina* (JSA1, 2, and 3 belong to *J. sabina* var. *Sabina*, and the remaining samples belong to *J. sabina* var*. balkanensis*). Red asterisk “*” indicates individuals not clustered with other individuals of the same species. Black asterisk “*” designates 100 bootstraps on the node. Lineages colors are the same as those used in the Bayesian inference.

The NJ phylogenetic tree was supported with high bootstraps on all nodes and provided a similar pattern to that of Bayesian clustering. This showed that all individuals of the same species clustered together except in three cases (Figure 1). Firstly, two individuals of *J. excelsa* from the Mrebbine population in north Lebanon belonged to the same evolutionary lineage of all *J. polycarpos* individuals. The second case concerned all *J. turcomanica* (=*J. polycarpos* var. *turcomanica*) individuals that were intermixed with *J. polycarpos*. The third case was observed for two individuals of *J. polycarpos* from the population Wadi El Njass in Lebanon collected in this study. These individuals clustered closely to the *J. seravschanica* lineage (Figure 1).

By taking *J. phoenicea* as an outgroup, the NJ tree revealed that all studied taxa belong to two major lineages. The first one contains all individuals of *J. chinensis*, *J. foetidissima*, *J. Sabina*, and *J. thurifera*. The second lineage includes all individuals of *J. excelsa, J. polycarpos, J. procera, J. seravschanica*, and *J. turcomanica*. In both major lineages, each species is clearly separated from the others, except for *J. polycarpos, J. turcomanica*, and *J. seravschanica.* The latter species show unclear phylogenetic boundaries, despite the high number of AFLP markers studied. The current topology of the phylogenetic tree suggests that one or several ancestral lineages of *J. thurifera, J. chinensis*, and *J. sabina* could have contributed to the origin of the hexaploid *J. foetidissima*.

### 3.3. Admixture Patterns between Juniperus Taxa

**Figure 2:**
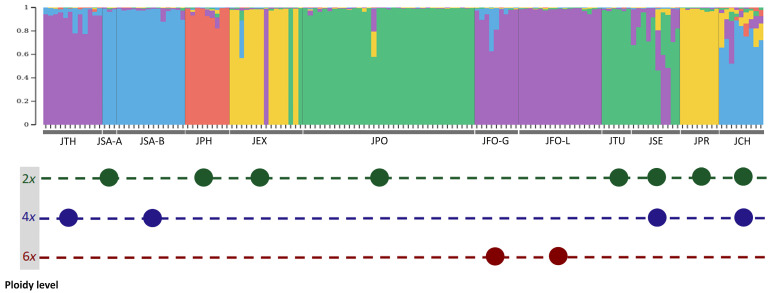
Plot of the Bayesian clustering analysis under the most likely solution, displaying five genetic groups (K = 5). Each vertical line represents one individual. Species abbreviations: JEX: *J. excelsa*, JPR: *J. procera*, JSE: *J. seravschanica*, JTU: *J. turcomanica*, JPO: *J. polycarpos*, JPH: *J. phoenicea*, JFO-G: *J. foetidissima* from Greece, JFO-L: *J. foetidissima* from Lebanon, JTH: *J. thurifera*, JCH: *J. chinensis*, and JSA-A: *J. sabina* var. *sabina*, and JSA-B: *J. sabina* var. *balkanensis*. Below, the ploidy level(s) reported for each taxon [7] is/are represented by a circle colored in green, blue, and dark-red for diploid, tetraploid, and hexaploid levels, respectively.

The Bayesian clustering analysis provided K = 5, 6, and 7 ancestral genetic clusters as the best fits for the present data. However, when six and seven genetic clusters were considered, one and two “empty” clusters, respectively, were clearly identified (clusters with only very low or null coancestry values across the whole sample;). We therefore focused only on K = 5 as the best and more realistic number of ancestral genetic clusters. Three solutions for K = 5 (Figure 2 and Figure S2) were identified on the basis of 50 runs. The most likely solution (Figure 2) held the highest probability (50%), followed by the less likely solutions A (36%) and B (14%) (Figure S2). In addition, the most likely solution (Figure 2) was the only one that displayed five truly distinct genetic groups, because solutions A and B each displayed one “empty” genetic group, with the fifth one being ghosted as part of the genetic identity of *J. chinensis* (genetic cluster represented by the yellow in Figure S2). For the aforementioned reasons, we based our interpretation in this study mainly on the most likely solution presented in Figure 2.

The Bayesian clustering therefore revealed that *J. phoenicea* clearly belongs to a separate genetic cluster. *Juniperus polycarpos*, *J. turcomanica*, and *J. seravschanica* were overwhelmingly assigned to the same cluster. However, individuals from *J. seravschanica* showed an admixed genome composition between the *J. polycarpos* cluster and the same cluster as *J. thurifera* and *J. foetidissima*. Both varieties of *J. sabina*, i.e., *J. sabina* var. *sabina* (2*n* = 2*x*) and *J. sabina* var. *balkanensis* (2*n* = 4*x*), belong to the same genetic cluster with no admixture pattern, except for two individuals of *J. sabina* var. *balkanensis*, which presented a slight level of admixture with the genetic cluster of *J. foetidissima* and *J. thurifera*.

*Juniperus excelsa* and *J. procera* belong to the same genetic cluster. Remarkably, three individuals of *J. excelsa* were purely assigned to two different genetic clusters, two individuals to the *J. polycarpos,* cluster and the remaining one to the *J. thurifera* and *J. foetidissima* cluster. Additionally, a third *J. excelsa* individual displayed a strong signal of genome coancestry with the *J. sabina* cluster.

*Juniperus chinensis* showed a clear trend of ancestry with the *J. sabina* cluster, in accordance with their evolutionary proximity shown on Figure 1. However, it also displayed a complex mosaic pattern of coancestry with the genetic clusters of *J. excelsa/J. procera* and *J. phoenicea.*

The hexaploid *J. foetidissima* and the tetraploid *J. thurifera* were clearly assigned to the same genetic cluster. This was also very clear in solution B (only four genetic clusters, see above) and only partly confirmed in solution A (Figure S2).

In the most likely solution (Figure 2), *J. thurifera* showed a slight signal of genetic admixture, with two individuals displaying genome coancestry (21% and 22%) with the same genetic cluster as *J. sabina* (a trend that was again more obvious in solution A but not in solution B). Similarly, these two genetic clusters were shown in the genetic ancestry of two *J. foetidissima* individuals from Greece (18% and 36%). In contrast, the population of *J. foetidissima* from Lebanon did not show any admixture signals in its ancestry.

## 4. Discussion

AFLP markers have been shown to display strong phylogenetic signals among closely related species [17,18,19,20,21]. Homoplasy or comigration of DNA fragments generated from different loci [29] is a potential challenge to the phylogenetic reconstruction based on AFLP markers. However, it was demonstrated to be a minor issue for studies dealing with closely related species [16], because homoplasy is expected to be less frequent. Additionally, the dominant nature of AFLP markers (the absence of a given amplified fragment being recessive) was also pointed out as a potential problem in assessing the genetic distance between a polyploid and each of its parents because the level of genotyping uncertainty increases with the ploidy level [36]. However, as noted by these authors, this should not inhibit the detection of hybridizations. Numerous studies have confirmed the power of AFLP markers in detecting interspecific hybridization (e.g., [23,24,37,38,39]). Altogether, the 1022 loci produced in this study allowed a powerful discrimination between the studied species and the identification of several individuals that could be considered as progenies of interspecific hybridization.

### 4.1. Genetic Delimitation of Diploid Juniperus Taxa

Globally, the Bayesian clustering approach and phylogenetic analysis produce largely convergent results. Among the studied taxa, *J. phoenicea*, *J. excelsa*, *J. procera*, *J. polycarpos*, and *J. turcomanica* are exclusively diploid [7]. The results presented above showed that *J. phoenicea* is clearly an independent evolutionary lineage, consistent with available phylogenetic analysis inferring this species to the base of all ca. 60 species of the *Sabina* section [8].

Our data also showed that *Juniperus excelsa* and *J. procera* are genetically strongly related to each other, a result that is congruent with previous phylogenetic analyses based on ITS and four chloroplast genes [7,8]. Unexpectedly, two individuals of *J. excelsa* from the Mrebbine population in north Lebanon belong to the same genetic group as *J. polycarpos* in the Bayesian clustering and were located within the cluster of *J. polycarpos* in the NJ tree, indicating a possible misidentification of these individuals. Due to the morphological uniformity between the two species, *J. polycarpos* was discovered in Lebanon only few years ago based on genetic investigations [8,25,40,41]. Indeed, the separation between *J. polycarpos* and *J. excelsa* has been demonstrated solely based on microsatellite (SSR), nrDNA (ITS), and chloroplast sequences [40,41] and never on the basis of morphological traits. Therefore, along with the mentioned markers, the AFLP markers provided a very clear and strong separation between these two species and represent a future promising cost-effective method for a heuristic geographic screening of these taxa. On the other hand, the AFLP markers failed to differentiate between *J. polycarpos* and *J. turcomanica* (=*J. polycarpos* var. *turcomanica*). In the present study, both species belonged to the same cluster without a clear delimitation between them (Figure 1 and Figure 2). Congruently, previous studies using a few nuclear (low copy genes and ITS) and four chloroplast sequences [41,42] showed no clear separation between these two taxa. Therefore, based on the current and aforementioned studies, we propose considering *J. polycarpos* and *J. turcomanica* as a species complex. Deep phylogenomic analyses must be conducted to obtained a clear understanding of this complex species, notably using NGS techniques such as GBS [43] and Hyb-Seq [44].

### 4.2. Potential Origin of the Tetraploid Junipers

*Juniperus sabina*, *J. chinensis*, and *J. seravschanica* have two cytotypes (2*n* = 2*x* and 4*x*) [7], whereas *J. thurifera* was the only studied species that is exclusively tetraploid. In this study, we analyzed two varieties of *J. Sabina*: the diploid *J. sabina* var. *sabina* (population: Austria, Alps) and the tetraploid *J. sabina* var. *balkanensis* (populations: Bosnia-Herzegovina and Croatia). Both varieties clustered in the same branch in the NJ tree and belonged to the same genetic group in the Bayesian clustering analysis. Only very few events of admixture were found for the tetraploid variety, and this result could be interpreted as a signal of shared ancestral polymorphism. Recently, *J. sabina* var. *balkanensis* has been described as a hybrid between the diploid variety of *J. sabina* and the tetraploid *J. thurifera*, since it holds the nuclear nrDNA (ITS) sequence of *J. sabina* var. *sabina* and the chloroplast haplotype (based on four genes) of *J. thurifera* [22,32,33]. These studies failed to uncover any evidence of a contribution of *J. thurifera* to the nuclear marker (ITS) of *J. sabina* var. *balkanensis*, in agreement with the AFLP in the present study despite the large number of loci. Therefore, it is likely that, after the ancient hybridization between a female *J. sabina* and a male *J. thurifera*, at least most of parental genetic nuclear material from *J. thurifera* was removed in the lineage of *J. sabina* var. *balkanensis*. This could have been achieved by several generations of backcross to the female *J. Sabina*, as described in one of the parsimonious hypothetical polyploidization pathways presented by Farhat et al. [12]. However, meiotic disorders may have helped in accelerating this process of elimination of one parental genetic material from the genome of the interspecific hybrid. Recently, interspecific hybridization was detected between the *J. sabina* (2*n* = 2*x*) and *J. thurifera* (2*n* = 4*x*) present in sympatric populations in France (Alps) and Spain (in two populations), giving rise to triploid and possibly tetraploid individuals [23,24]. Interestingly, male triploid hybrids examined in the French Alps population showed well-conformed and potentially fertile pollen [23], which might reflect the fertility of the triploid hybrids. Consequently, the presence of fertile triploid hybrids might engender a better establishment of the polyploid toward a more stable ploidy level. These latter findings provide more support for the hybrid origin of the tetraploid *J. sabina* var. *balkanensis* involving the parental species *J. thurifera* and *J. sabina*.

*Juniperus chinensis* showed a complex genetic pattern. However, interpretation of this pattern should be very cautious. It could be partly due to the fingerprint of shared ancestral polymorphism and/or to uncertainties in ancestry inferences because of the constrained and limited number of ancestral genetic cluster of the solutions retained after Bayesian clustering (five or four genetic clusters in this analysis). Nevertheless, our results strongly underline the strong coancestry of *J. chinensis* individuals with *J. sabina* individuals. In addition, *J. chinensis* was located in the NJ tree on the sister branch of *J. sabina*. This is compatible with a previous analysis showing that *J. sabina* and *J. chinensis* are phylogenetically closer to each other than the remaining taxa examined in our study [8]. Additionally, the complex admixed genetic pattern of *J. chinensis* found in this study might be the consequence of plausible introgression events from neighboring taxa. *Juniperus chinensis* is currently distributed in China and Japan, far from all the species analyzed in this study, except for *J. sabina* var. *sabina*, which has a wide distribution reaching China (Figure S3) [8]. Therefore, if the observed complex genetic pattern in *J. chinensis* was produced by introgression events, it might implicate historically dated events during which the geographical distribution of the studied species was possibly closer than that observed currently.

The revealed pattern of genetic admixture of the genome of most *J. seravschanica* individuals between the genetic groups of *J. polycarpos*/*J. turcomanica* and *J. foetidissima/J. thurifera* suggests that this species is a hybrid. This result is in accordance with the phylogenetic closeness previously revealed between *J. seravschanica* and *J. foetidissima* [8]. Moreover, it has lately been suggested based on nrDNA (ITS) and four chloroplast regions that *J. seravschanica* obtained the chloroplasts from an ancestor of *J. foetidissima*/*J. thurifera* through an ancient chloroplast capture [45], meaning that hybridization has occurred in the past between these two lineages (*J. seravschanica* and *J. foetidissima*/*J. thurifera*). Interestingly, the studied populations of *J. seravschanica* were previously identified as tetraploid [7]. Therefore, the AFLP data suggest that tetraploid individuals of *J. seravschanica* issued from an interspecific hybridization event, and the inferred admixed status of the genome of some of *J. seravschanica* individuals may testify to their allopolyploid nature. Based on the admixed pattern revealed in this study and the chloroplast capture analysis of Adams [45], we propose that the *J. polycarpos*/*J. turcomanica* lineage has contributed maternal parent and the *J. foetidissima*/*J. thurifera* lineage has contributed the paternal parent of tetraploid *J. seravschanica*. In addition, the strong admixture level shown by a few *J. seravschanica* individuals (ca. 50% of the admixture) (Figure 2) may also be the consequence of still ongoing genetic introgression. Indeed, the current geographical distributions of *J. foetidissima*, *J. polycarpos*, *J. turcomanica*, and *J. seravschanica* are currently overlapping. More in-depth analysis using large and genome-wide DNA sequence data should be conducted with a more extensive and more geographically representative sample to look further into these hypotheses. Especially, the sympatry zones of *J. seravschanica* with parental species candidates would be of prime interest from this perspective. In contrast, *J. thurifera* is currently very distant from the aforementioned species (Figure S3). Therefore, if we assume that the introgression event leading to the formation of *J. seravschanica* was recent, the geographical distribution of these species supports *J. foetidissima* rather than *J. thurifera* as the paternal parent. In this potential scenario on the origin of tetraploid *J. seravschanica*, the fertilization between a reduced gamete from *J. foetidissima* (*n* = 3*x*) and a reduced gamete from the diploid *J. polycarpos/J. turcomanica* (*n* = *x*) would directly produce tetraploid progeny.

*Juniperus thurifera* belongs to the same genetic cluster as *J. foetidissima* without a significant admixture pattern, with the exception of two individuals from Greece, in which a large part of the genome (>20%) was assigned to the *J. sabina* cluster (Figure 2). A similar case was previously found in a population from the French Alps, where *J. thurifera* and *J. sabina* were present in sympatry and where triploid hybrids producing well-conformed pollen grains were discovered [23]. In this mixed population, individuals morphologically identified as *J. thurifera* were genetically found to have an admixed genetic pattern between *J. thurifera* and *J. sabina*. Therefore, Farhat et al. [23] designated these individuals as tetraploid progenies of hybrids originating from the triploid hybrids between *J. thurifera* and *J. sabina*. It is therefore possible that *J. thurifera* individuals showing a slightly admixed genetic pattern with *J. sabina* in the present study are also the result of genetic introgression from *J. sabina*. Previous phylogenetic analyses showed that *J. thurifera* and *J. foetidissima* were separated by sister branches [7,8], which is not the case in our NJ phylogenetic tree because *J. thurifera* appears to be closer to *J. sabina* (Figure 1). The existence among our studied samples of *J. thurifera* individuals genetically introgressed by genetic material from *J. sabina* may account for this discrepancy. Altogether, our results show no clear evidence of hybridization in the origin of the tetraploid *J. thurifera* but confirm that *J. thurifera* harbors signs of genetic introgression from *J. sabina* in its genome due to the existence of recent gene flow between the species.

### 4.3. Insights into the Origin and Hypothetical Hexaploidy Pathway in J. foetidisima

Despite the rarity of high ploidy levels in conifers, *J. foetidissima* was determined as a hexaploid species [7], which is the highest ploidy level reported in this group. *Juniperus foetidissima* and *J. thurifera* showed a clear genome assignment to the same ancestral genetic cluster, different from all remaining species except for *J. seravschanica* (see above). This observation and the fact that *J. thurifera* is tetraploid suggest that the ancestor of *J. foetidissima* belongs to the lineage of *J. thurifera*. Interestingly, most of the studied individuals of *J. foetidissima* from Greece presented a significant part of their genome assigned to the genetic group of *J. sabina*, similar to *J. thurifera* but in contrast to the individuals from Lebanon.

**Figure 3:**
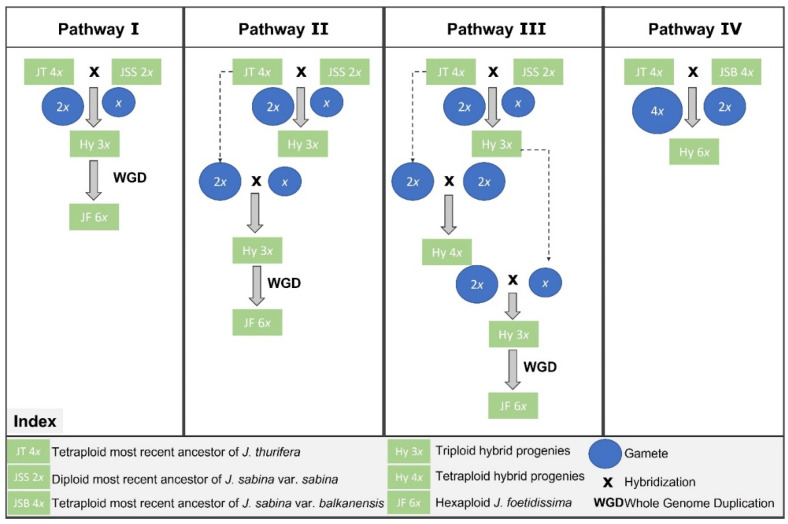
Simplified schematic representation of four hypothetical parsimonious pathways for the formation of the hexaploid *J. foetidissima*.

For a better understanding of the possible origins of *J. foetidissima* based on the currently available data, we illustrate four simplified schematic pathways in Figure 3 summarizing four hypothetical parsimonious pathways of this hexaploid formation. One of the hypotheses is that *J. sabina* and *J. thurifera* were both involved in the origin of *J. foetidissima*. This hypothesis is supported by the existence of interspecific hybrids between those two species reported in several populations in France and Spain [23,24]. Interestingly, in these populations, the ploidy level of hybrids was either triploid or tetraploid. Both triploid and tetraploid hybrids may have been involved in the genesis of the hexaploid lineage through several pathways (Figure 3, pathways I, II, and III). The first pathway could be the union between a 2*x* gamete from the tetraploid *J. thurifera* ancestor and an *x* gamete of the diploid *J. Sabina* ancestor that gave rise to triploid progeny (Figure 3, pathway I). A whole-genome duplication (WGD) in triploid hybrids allows us to restore cytogenetic stability and full fertility and is therefore selectively advantageous (Figure 3, pathway I). According to this pathway, the expected genomic contribution of *J. sabina* to the hexaploid genome of *J. foetidissima* would be one-third at the beginning of the process, which is in accordance with the admixed genome pattern of only a few individuals from Greece in our study. However, different mechanisms such as high rates of nonhomologous association, homologous recombination, homologous chromosome replacement, and bias in chromosomal segregation may produce diverse genetic combination in the progenies of newly formed polyploid individuals with variable contribution of genomes from both parental species (e.g., [46]). Additionally, selection can then favor or eliminate certain combinations, or drift processes and the founder effect along pathways of dispersal can lead to the random fixation of certain genetic combinations. All those processes could have happened in the case of the evolution of *J. foetidissima* in the eastern part of the Mediterranean region.

Alternative pathways are illustrated in Figure 3 (pathways II and III), following possibly similar scenarios as discovered in the French Alps and Spain [23,24]. The triploid generated after the cross between ancestors of *J. thurifera* (*n* = 2*x*) and *J. sabina* (*n* = *x*) would, in turn, produce gametes with three ploidy levels: *n* = *x*, 2*x*, and 3*x*. It is very unlikely that the triploid gamete would interfere in this scenario with the ancestor of *J. thurifera* (2*n* = 4*x*) because it would have resulted in pentaploid progenies. Therefore, we discard this possibility. In the first case, the cross of the (*n* = *x*) gamete of the neo-triploid hybrid with (*n* = 2*x*) of *J. thurifera* ancestor would produce another triploid hybrid that would duplicate its genome through a WGD reaching the hexaploid level (Figure 3, pathway II). In the second case, the diploid gamete (*n* = 2*x*) of the neo-triploid hybrid might unify with the *J. thurifera* ancestor (*n* = 2*x*), giving rise to tetraploid level progenies, as found in the French Alps (Figure 3, pathway III). A further hybridization between the triploid and tetraploid progenies (2*n* = 3*x* and 2*n* = 4*x*) may give rise to triploid hybrids (among other ploidy levels), followed by a WGD reaching the hexaploid level. In both pathways II and III, the original contribution of the genome of *J. sabina* to the genome of *J. foetidissima* may widely vary from zero to one-third.

On the other hand, if the hybridization between the ancestors of *J. thurifera* and *J. sabina* happened before the WGD in the *J. thurifera* lineage, the only scenario to reach a hexaploid level after hybridization would be through the contribution of “unreduced gametes” in one of the parents. This scenario would generate triploid hybrids followed by WGD. However, this hypothesis is not very plausible because the polyploidy event in *J. thurifera* was already estimated to be very old (paleo-tetraploid) based on cytological evidence [11].

All of the hypotheses discussed above suggest the diploid *J. Sabina* ancestor as one of the contributors to the *J. foetidissima* genome. The alternative possible hypothesis would involve both lineages of the tetraploid *J. thurifera* and the tetraploid *J. sabina* var. *balkanensis* (Figure 3, pathway IV). In this hypothesis, the fertilization between an unreduced gamete of *J. thurifera* (*n* = 4*x*) and a reduced gamete of the tetraploid *J. sabina* var. *balkanensis* (*n* = 2*x*) would directly result in hexaploid progenies. The expected original genetic contribution of *J. sabina* var. *balkanensis* in the hexaploid progeny would thus be one-third, in agreement with the admixed pattern shown in some individuals of *J. foetidissima* from Greece. The same mechanisms discussed above and the removal of most or all of the contribution of the *J. sabina* genome (pathway I) would result in the observed genetic pattern of *J. foetidissima* from Lebanon. There are three arguments in favor of this hypothesis, which may give it an advantage over the previously proposed origins. First, on the genetic level, the expected and observed genetic patterns of *J. foetidissima* (Greece) are congruent as explained above. Second, the meiotic level showed the possibility of producing well-formed (potentially fertile) unreduced pollen in *J. thurifera* from the French Alps [23]. The third is the geographical distribution of the parental species. Currently, the geographical ranges of the parental lineages *J. thurifera* and *J. sabina* var. *balkanensis* do not overlap. Nevertheless, the fact that *J. sabina* var. *balkanensis* originated from a hybridization between the diploid *J. sabina* and the tetraploid *J. thurifera* (Figure S3) implies that during the previous period, they would have had overlapped geographical distributions.

In our study, we focused on identifying the potential lineages of *J. foetidissima*, which we narrowed down to *J. thurifera* and *J. sabina*, including two of its varieties: *J. sabina* var. *sabina* (2*n* = 2*x*) and *J. sabina* var. *balkanensis* (2*n* = 4*x*). However, to gain a more comprehensive understanding of the origin of *J. foetidissima*, further evidence and insights are necessary. Ideally, a broader range of populations would provide informative genetic patterns and reveal the influence of the *J. sabina* lineage in the genome of *J. foetidissima*. It is important to determine if the genetic patterns of these populations are comparable to those found in the populations studied in Lebanon and Greece or if a potential third lineage could complete the understanding of the origin and evolution of the hexaploid *J. foetidissima*. It would also be plausible that the common ancestor of *J. thurifera/J. foetidissima* entered the WGD events in an autopolyploidy scenario, giving rise to two lineages, the first of which was the tetraploid “*Thurifera*” lineage that conquered the western part of Europe and the second was the hexaploid “*Foetidissima*” that occupied areas in the east. The key point to better identify the most probable polyploidy scenarios is to estimate the time period of the WDG events in *J. thurifera* and *J. foetidissima*. A genomic approach with complete sequencing is also clearly needed but is still highly challenging because of the very large size of the genomes of these species and the need to take into account, through sampling, the biogeographic variations in genomic diversity and possibly introgression patterns, the first drafts of which we obtained as a result. A genotyping-by-sequencing approach could be a fruitful intermediate method. Coalescence modeling based on large sequence data may help to infer the likelihoods and relative dating of major events of species admixtures that we hypothesized

## Figures and Tables

**Table 1:** Details on the studied populations of *Juniperus* species. *, material collected and used in the Ph.D. research of Bouchra Douaihy [25]. The ploidy level of each taxon was extracted from Farhat et al. [7]. The ploidy level appended by “i”, “ii”, or “iii” is the exact samples measured by Farhat et al. [7,12,23].

Taxon	Population	Somatic Ploidy Level	Country	Fresh/Dried Leaves	Number of Individuals	GPS Coordinates
*Juniperus excelsa*	Afqa *	2*x*	Lebanon	Fresh	4	N 34°4′25″, E 35°54′20″
	Barqa *		Lebanon	Fresh	1	N 34°11′48″, E 36°8′15″
	Devas Mountain, Agios Georgious Forest		Greece	Fresh	1	N 39°45′0.4716″, E 19°40′59.8944″
	Qammoua forest *		Lebanon	Fresh	5	N 34°29′34″, E 36°15′14″
	Mrebbine		Lebanon	Fresh	2	N 34°34′4.008″, E 36°7′33.996″
	Nebha, Alkedam, Wadi ghned, Arsal		Lebanon	Fresh	2	N 34°11′19.7952″, E 36°12′57.6684″
*J. chinensis*	Japan	4*x*^i^	Japan	Dried	3	N 35°01.44′, E 138°47.30′
	Lanzhou	2*x*, 4*x*	China	Dried	4	N 36°6′0″, E 103°43′59.88″
	Xian		China	Dried	2	N 34°08′50.2”, E 109°34′41.3”
*J. foetidissima*	Devas Mountain, Agios Georgious Forest	6*x*^i^	Greece	Fresh	9	N 39°45′0.4716″, E 19°40′59.8944″
	Hermel		Lebanon	Fresh	14	N 34°19′8.7528″, E 36°15′29.7792″
	Qammoua forest		Lebanon	Fresh	3	N 34° 30′ 44.388″, E 36°16′30.36″
*J. phoenicea*	Baćina	2*x*	Croatia	Fresh	9	N 43°05′12.6”, E 17°22′36.2”
*J. polycarpos*	Arsal *	2*x*	Lebanon	Fresh	17	N 34°4′57″, E 36°28′33.996″
	Azerbaijan	2*x*^i^	Azerbaijan	Dried	2	N 40°44′41.064″, E 47°35′19.14″
	Wadi El Njass *	2*x*	Lebanon	Fresh	8	N 34°19′49″, E 36°3′16″
	Wadi El Njass		Lebanon	Fresh	8	N 34°20′48″, E 36°5′17″
*J. procera*	Ethiopia	2*x*^i^	Ethiopia	Dried	4	N 9°1′59.9988″, E 38°23′60″
	near Abha	2*x*	Saudi Arabia	Dried	4	N 18°16′59.988″, E 42°21′0″
*J. sabina* var. *sabina*	Austria, Alps	2*x*^ii^	Austria	Dried	3	N 46°56′6″, E 11°2′20.4″
*J. sabina* var. *balkanensis*	Mts. Cvrsnica and Cabulja	4*x*^ii^	Bosnia-Herzegovina	Fresh	7	N 43°34′18.084″, E 17°30′39.888″
	Biokovo Mts.		Croatia	Fresh	7	N 43°19′31.1808″, E 17°2′57.0228″
*J. seravschanica*	Kazakhstan	4*x*^i^	Kazakhstan	Dried	2	N 42°24′31.788″, E 70°28′30″
	Kazakhstan		Kazakhstan	Dried	3	N 42°10′46.2″, E 70°20′0.816″
	Oman	2*x*, 4*x*	Oman	Dried	3	N 23°7′41.016″, E 57°36′9.288″
	Pakistan		Pakistan	Dried	2	N 30°13′0.012″, E 67°5′60″
*J. thurifera*	Alps	4*x*^iii^	France	Dried	5	N 44°43′7.986″, E 6°36′13.0392″
	Monegros region	4*x*^i^	Spain	Fresh	3	N 41°36′19.3428″, W 0°15′7.092″
	Southern Iberian central range		Spain	Fresh	4	N 40° 0′ 14.4936″, W 5°1′11.82″
*J. turcomanica*	Turkmenistan	2*x*^i^	Turkmenistan	Dried	2	N 38°25′7.212″, E 56° 58′ 48″
	Shahmirzad	2*x*	Iran	Dried	1	N 35°50′54.996″, E 53°26′24.216″
	Bajgirna		Iran	Dried	1	N 37°25′9.804″, E 58°32′0.204″
	Baladae		Iran	Dried	1	N 36°14′34.404″, E 51°50′20.4″
	Fasa		Iran	Dried	1	N 29°9′57.816″, E 53°40′7.788″

**Table 2:** Number of replicates for each selected species from: repetition type 1 (repetition from same DNA and independent restriction digests) and repetition type 2 (repetition from the same sample and different DNA extractions).

Species	Replicate Number of Repetition Type 1	Replicate Number of Repetition Type 2
*J. excelsa*	1	2
*J. polycarpos*	3	6
*J. foetidissima*	1	12
*J. seravschanica*	1	0
*J. turcomanica*	1	0
*J. chinensis*	1	0
*J. procera*	1	0
*J. sabina*	1	2
*J. thurifera*	1	0
*J. phoenicea*	1	0

**Table 3:** Details of the AFLP adapters and primers used in this study.

	CODE	Primer (5′-> 3′)	Length	GC (%)	Tm	Label
Adapter	EcoRI-adapter L	CTCGTAGACTGCGTACC	17	58.8	55.2	No label
	EcoRI-adapter S	AATTGGTACGCAGTC	15	46.7	45.1	No label
	Tru1I-adapter L	GACGATGAGTCCTGAG	16	56.3	51.7	No label
	Tru1I-adapter S	TACTCAGGACTCAT	14	42.9	40	No label
Preamplification	EcoRI-PA	ACTGCGTACCAATTCA	16	43.8	46.6	No label
	Tru1I-PA	GATGAGTCCTGAGTAAC	17	47.1	50.4	No label
Amplification	EcoRI-1	ACTGCGTACCAATTCACG	18	50	53.7	FAM
	EcoRI-2	ACTGCGTACCAATTCACT	18	44.4	51.4	FAM
	EcoRI-4	ACTGCGTACCAATTCACC	18	50	53.7	PET
	Tru1I-1	GATGAGTCCTGAGTAACTA	19	42.1	52.4	No label
	Tru1I-2	GATGAGTCCTGAGTAACTG	19	47.4	54.5	No label
	Tru1I-3	GATGAGTCCTGAGTAACAG	19	47.4	54.5	No label
	Tru1I-4	GATGAGTCCTGAGTAACTT	19	42.1	52.4	No label
Amplification primers combinations	EcoRI-1/Tru1I-1					
	EcoRI-1/Tru1I-3					
	EcoRI-2/Tru1I-4					
	EcoRI-4/Tru1I-2					

**Table 4:** Proportion (PLP) of polymorphic loci (%) generated by AFLP from the four primer combinations of the studied *Juniperus* species. The red asterisk (*) represents the highest PLP in a primer combination, and the black asterisk (*) represents the lowest PLP.

Species	Primers EcoRI-1/Tru1I-1	Primers EcoRI-1/Tru1I-3	Primers EcoRI-2/Tru1I-4	Primers EcoRI-4/Tru1I-2
*J. chinensis*	68.5 *	58.5 *	73.1 *	59.9 *
*J. excelsa*	46.8	48.1	60.6	44.2
*J. foetidissima*	50.9	42.2	48.5	41.6
*J. phoenicea*	39.1	42.5	49.8	37.2
*J. polycarpos*	38.4 *	36.9	50.8	32
*J. procera*	40.4	38.7	50.8	32
*J. sabina*	49.1	41.1	53.2	37.2
*J. seravschanica*	56.8	46	56.6	43.9
*J. thurifera*	44.2	36.2 *	43.8 *	32.7
*J. turcomanica*	39.9	37.6	54.5	31.2 *

## Data Availability

The data presented in this study are available on request from the corresponding authors.

## Supplementary Materials

The following are available online at https://www.mdpi.com/article/10.3390/life13071479/s1, Figure S1. Plot of the deviance information criterion (DIC) indicating the best genetic group (K) values for the studied AFLP markers data. Figure S2. Plot of the Bayesian clustering analysis under the less likely solutions A and B under the genetic group K = 5. Figure S3. Geographical distribution of the studied *Juniperus* taxa extracted from (Adams 2014 and Farhat et al., 2019, [7,8]).
