# Supplementary material for: Allopolyploidy: An Underestimated Driver in Juniperus Evolution
Source: Life (Basel). 2023 Jun 30;13(7):1479. doi: 10.3390/life13071479 (PMC10381917; doi:10.3390/life13071479)
Supplement: Supplementary file 1 [file life-13-01479-s001.zip › life-2303147-supplementary.pdf]

Supplementary Material

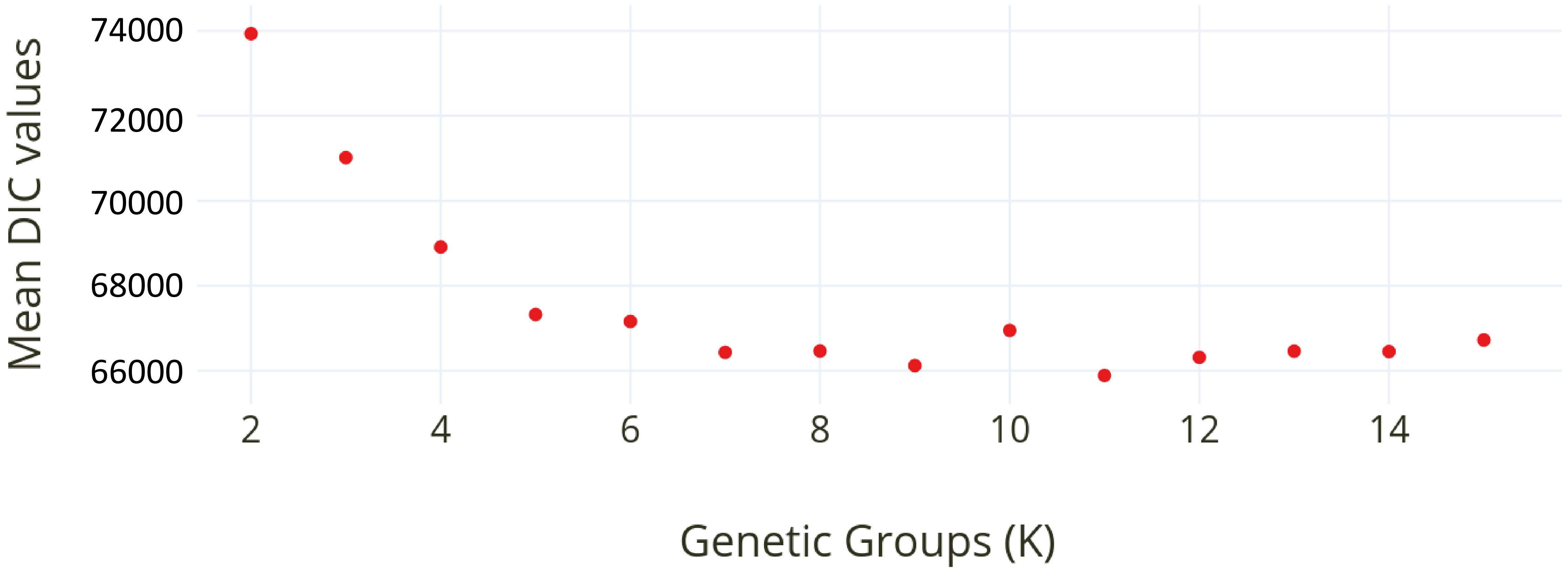

Figure S1. Plot of the Deviance Information Criteria (DIC) indicating the best genetic group (K) values for the studied AFLP markers data

Solution-A

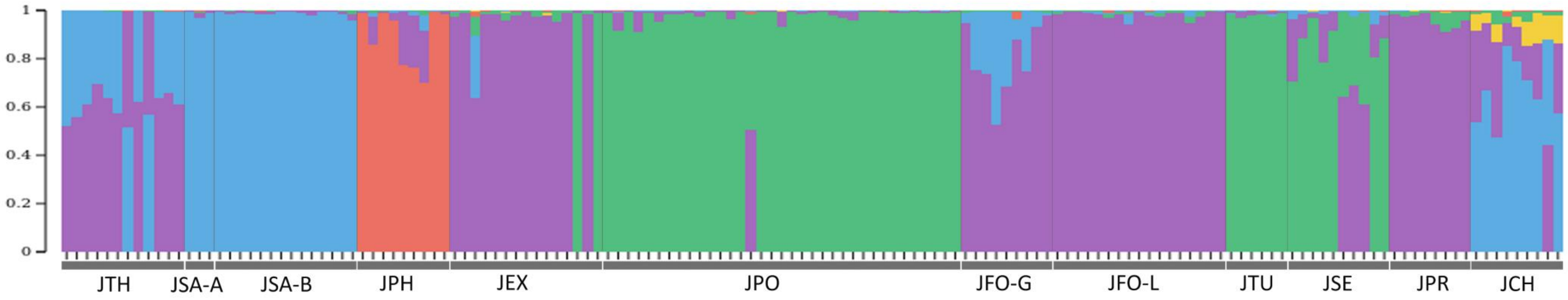

Solution-B

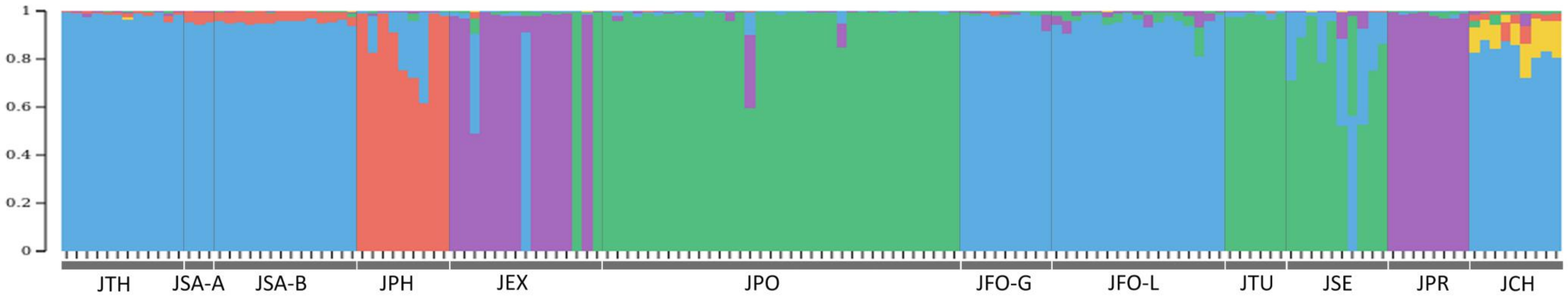

Figure S2. Plot of the Bayesian clustering analysis under the solutions A and B under the genetic group K = 5.

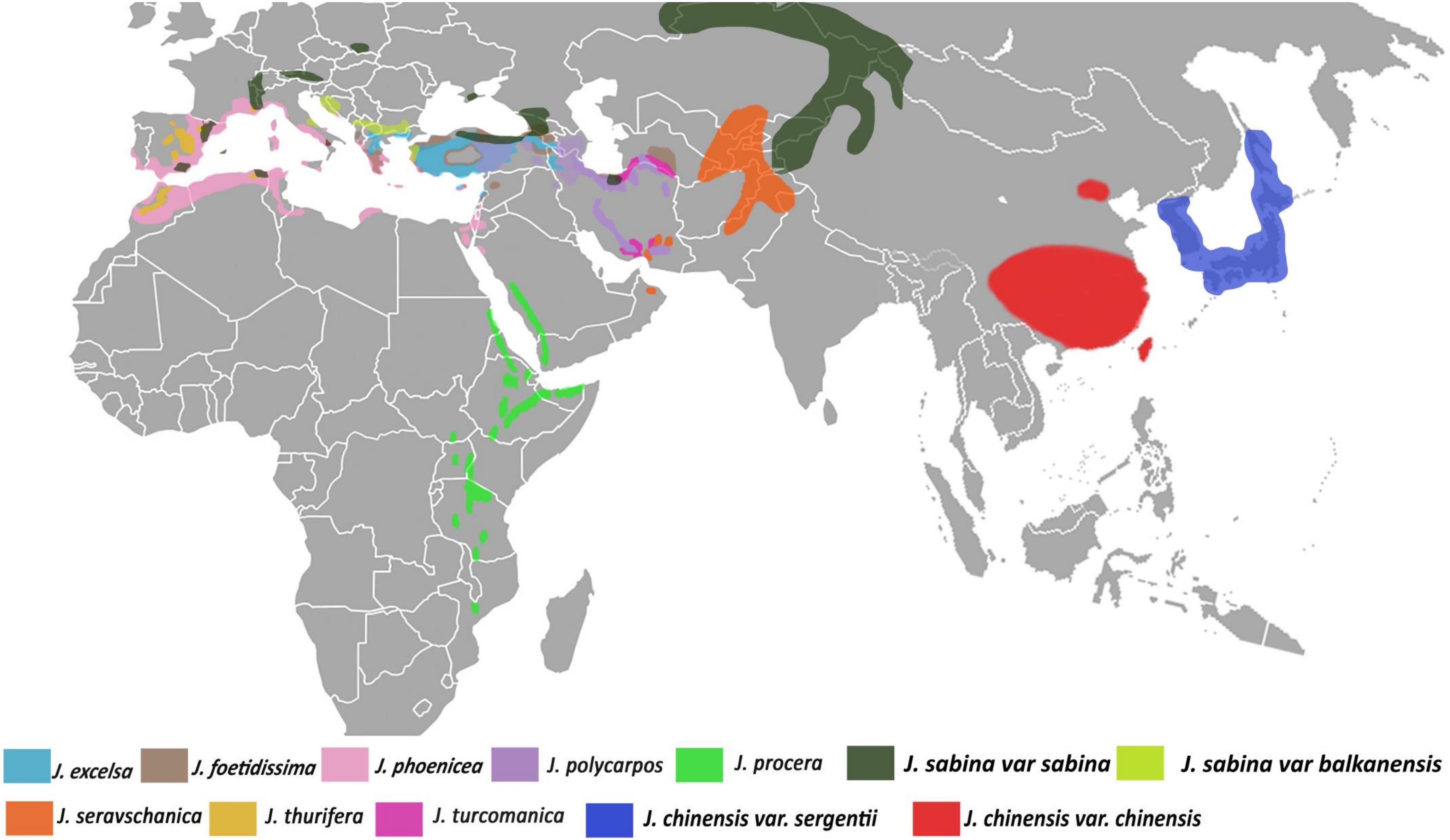

Figure S3. Geographical distribution of the studied *Juniperus* taxa extracted from (Adams 2014 and Farhat et al 2019).
